# Partial Protection against Porcine Influenza A Virus by a Hemagglutinin-Expressing Virus Replicon Particle Vaccine in the Absence of Neutralizing Antibodies

**DOI:** 10.3389/fimmu.2016.00253

**Published:** 2016-06-30

**Authors:** Meret E. Ricklin, Nathalie J. Vielle, Sylvie Python, Daniel Brechbühl, Beatrice Zumkehr, Horst Posthaus, Gert Zimmer, Artur Summerfield

**Affiliations:** ^1^Institute of Virology and Immunology, Mittelhäusern, Switzerland; ^2^Vetsuisse Faculty, Institute for Animal Pathology, University of Bern, Bern, Switzerland; ^3^Department of Infectious Diseases and Pathobiology, Vetsuisse Faculty, University of Bern, Bern, Switzerland

**Keywords:** influenza virus, hemagglutinin, VRP vaccine, pig, opsonization, neutralization, CD4 T cells

## Abstract

This work was initiated by previous reports demonstrating that mismatched influenza A virus (IAV) vaccines can induce enhanced disease, probably mediated by antibodies. Our aim was, therefore, to investigate if a vaccine inducing opsonizing but not neutralizing antibodies against the hemagglutinin (HA) of a selected heterologous challenge virus would enhance disease or induce protective immune responses in the pig model. To this end, we immunized pigs with either whole inactivated virus (WIV)-vaccine or HA-expressing virus replicon particles (VRP) vaccine based on recombinant vesicular stomatitis virus (VSV). Both types of vaccines induced virus neutralizing and opsonizing antibodies against homologous virus as shown by a highly sensitive plasmacytoid dendritic cell-based opsonization assay. Opsonizing antibodies showed a broader reactivity against heterologous IAV compared with neutralizing antibodies. Pigs immunized with HA-recombinant VRP vaccine were partially protected from infection with a mismatched IAV, which was not neutralized but opsonized by the immune sera. The VRP vaccine reduced lung lesions, lung inflammatory cytokine responses, serum IFN-α responses, and viral loads in the airways. Only the VRP vaccine was able to prime IAV-specific IFNγ/TNFα dual secreting CD4^+^ T cells detectable in the peripheral blood. In summary, this work demonstrates that with the virus pair selected, a WIV vaccine inducing opsonizing antibodies against HA which lack neutralizing activity, is neither protective nor does it induce enhanced disease in pigs. In contrast, VRP-expressing HA is efficacious vaccines in swine as they induced both potent antibodies and T-cell immunity resulting in a broader protective value.

## Introduction

Influenza A virus (IAV) is a negative-sense RNA virus of the family *Orthomyxoviridae*. The genome is composed of 8 gene segments coding for 11 gene products. The natural reservoir for IAV is wild waterfowl, but distinct IAV lineages also exist in pigs, horses, dogs, sea mammals, bats, and humans (1–3). IAV from animal origin have zoonotic potential and may adapt to human hosts. The enormous genetic plasticity of these viruses facilitates this process.

The hemagglutinin (HA) is the most abundant and important antigen of the IAV envelope. It induces virus-neutralizing antibodies that interfere with either receptor-binding membrane fusion or viral egress (4, 5). Therefore, HA is the prime antigen of influenza virus vaccines. However, HA is subject to antigenic drift caused by point mutations and antigenic shift caused by segment exchange, and both processes result in mutant viruses that may escape pre-existing immunity. For this reason, licensed inactivated virus vaccines, which induce immunity mainly through their ability to induce neutralizing antibodies, must match the currently circulating field viruses. Of additional concern is that in some cases pre-existing immunity against heterologous IAV strains can even lead to enhanced disease, as observed in human and pigs (6–10). A study in humans suggested that enhanced disease is associated with immune complexes involving non-neutralizing antibodies (11). However, the role of individual viral proteins in this process has not been investigated.

Consequently, using an HA-expressing vesicular stomatitis virus (VSV) vector and a whole inactivated virus (WIV) vaccine, this study addressed if vaccines selected to induce opsonizing but not neutralizing antibodies against a heterologous H1N1 porcine IAV strain would confer enhanced disease or protection. While we did not find any evidence for enhanced disease, pigs were partially protected if they had received the VSV vaccine, which induced both opsonizing and IAV-specific dual-functional CD4 T helper cells.

## Materials and Methods

### Cells

BHK-21 cells were (ATCC, Manassas, VA, USA) grown in Earle’s minimal essential medium (MEM) (Life Technologies, Zug, Switzerland) supplemented with fetal bovine serum (FBS) (Biowest, Nuaillé, France). BHK-G43, a transgenic BHK-21 cell clone expressing the VSV G protein, was maintained, as described previously (12). Madin–Darby canine kidney (MDCK; ATCC) cells were propagated in MEM supplemented with 10% FBS, non-essential amino acids (Life Technologies), and 1 mM sodium pyruvate (Life Technologies).

### Viruses

The viruses used are summarized in Table 1. All IAV were propagated in 10-day-old embryonated specific-pathogen-free (SPF) chicken eggs incubated for 2 days at 37°C. IAV were titrated on confluent MDCK cells in presence of 1 μg/ml of acetylated trypsin (Sigma-Aldrich). After 24 h, cells were fed by addition of new medium, and 48 h post-infection (p.i.), the cells were fixed with 4% paraformaldehyde (Polysciences, Warrington, PA, USA). The plates were stained with anti-NP antibody (HB-65, ATCC) in saponin (Sigma-Aldrich), followed by horseradish peroxidase-conjugated goat anti-mouse antibody (Dako, Baar, Switzerland), and a final color reaction with 3-amino-9-ethylcarbazole (AEC, Sigma-Aldrich). Titers were calculated using the Reed and Muench formula.

**Table 1:** **Viruses used in this study**.

Full name	Subtype	Short name	Source
A/swine/Bakum/IDT1769/2003	H3N2	IDT1769/03	FLI
A/swine/Bakum/R325/2009	H3N2	R325/09	FLI
A/swine/Bakum/R757/2010	H1N2	R757/10	FLI
A/swine/Germany-BB/siv-leipz11308/09	H1N1	Leipzig/09	FLI
A/swine/Belzig/2/01	H1N1	Belzig/01	FLI
A/swine/Belgium/1/98	H1N1	Belgium/98	UGent
A/swine/Bülow/1/81	H1N1	Bülow/81	UGent
A/California/04/2009	H1N1	California/09	HUG
A/swine/Gent/7625/99	H1N2	Gent/99	UGent
A/swine/Flanders/1/98	H3N2	Flanders/98	UGent
A/New Caledonia/20/99	H1N1	NC/99	HUG
A/swine/Wessin/2009	H1N1	Wessin/09	FLI
A/swine/Iowa/15/30	H1N1	Iowa/30	ATCC
A/swine/Germany/R248/2010	H1N1	R248/10	FLI

### Plasmids

The cDNAs encoding HA or NA of H1N1 A/swine/Belzig/2/01 (Belzig/01) were kindly provided by Drs. Jürgen and Olga Stech (Friedrich-Loeffler-Institute, Riems, Germany) (13). The open reading frames of IAV antigens were amplified by PCR and cloned into the pVSV* plasmid using the *Mlu*I and *Bst*EII endonuclease restriction sites placed upstream and downstream of the fourth transcription unit of the VSV genome (14). To facilitate titrations, a cDNA encoding enhanced green fluorescent protein (eGFP) was inserted into an additional transcription unit located in the intergenic region between the original G and L genes. The resulting plasmids were designated pVSV*ΔG(H1) and pVSV*ΔG(N1), in which the asterisk denotes the presence of the eGFP gene and ΔG indicates the absence of the glycoprotein G gene.

### Generation of VRP

Virus replicon particles (VRP) lacking the envelope glycoprotein G were generated, as described previously (14, 15). Briefly, BHK-G43 cells were infected with recombinant MVA-T7 expressing T7 RNA polymerase (16) and subsequently transfected with a plasmid carrying a VSV anti-genomic cDNA, along with three plasmids encoding the VSV proteins N, P, and L. All genes were placed under the control of the T7 promoter. Expression of the VSV G protein was induced in BHK-G43 cells by adding 10^−9^M mifepristone (Sigma-Aldrich) to the culture medium. At 24 h post-transfection, the cells were trypsinized and seeded into T75 cell culture flasks (Corning B.V., Amsterdam, The Netherlands), along with an equal number of fresh BHK-G43 cells. The cells were incubated at 37°C for 24 h in the presence of mifepristone. The cell culture supernatant was clarified by low-speed centrifugation and passed through a 0.20-μm pore-size filter. Recombinant viruses were propagated on mifepristone-induced BHK-G43 cells to generate VRP, which were titrated on BHK-21. Infectious titers were expressed as fluorescent focus-forming units (FFU) per milliliter.

### Animal Experiments

The animal experiments were performed according to local law and were approved by the Cantonal Ethical Committee for Animal Experiments (BE 07-12). In total, 31 healthy 7-week-old Swiss Large White pigs (18 castrated males and 13 females) from our SPF breeding facility were used. Animals were housed in groups of ≥3 inside the containment facility of the Institute for Virology and Immunology (IVI) representing a BSL3-Ag facility. Prior to the infection, they were allowed 1 week of adaptation to the new environment.

To produce protein-specific antisera, two pigs per vaccine were immunized with VSV*ΔG (negative control), VSV*ΔG(H1), and VSV*ΔG(N1) into the gluteal and deltoideal muscle with a dose of 10^8^ TCID_50_ in a volume of 4 ml, followed by a boost 4 weeks later. Two additional piglets were vaccinated with binary ethyleneimine (BEI) inactivated Belzig Virus (WIV) composed of 5.3 × 10^6^ TCID_50_ in a volume of 4 ml, formulated with 15% of Montanide 25VG (kindly donated by Seppic, Puteaux, France). The selection of this adjuvant was based on its known potency to induce antibody responses with inactivated virus preparations. Animals were bled weekly, and sera were frozen at −20°C.

For the vaccination/challenge experiment, animals were vaccinated in groups of five with VSV*ΔG, VSV*ΔG(H1), VSV*ΔG(N1), or WIV, as described earlier. After 4 weeks, an identical booster vaccination was done. Animals were examined clinically daily, and blood was taken once a week. Three weeks post-boost, all pigs were challenged intratracheally with 7 × 10^6^ TCID_50_ of H2N1 A/swine/Bakum/R757/2010 (R757/10) under general anesthesia with 0.5 mg/kg Midazolam (Roche, Reinach, Switzerland) and 10 mg/kg Ketamin (Provet, Lyssach, Switzerland). After challenge, all animals were clinically examined daily, including measuring body temperature, and assessing awareness, appetite, manure excretion, breathing, coughing, skin color, and gait. Blood and oronasal swabs were taken daily. After 24 h, each animal was bronchoscopied, and bronchoalveolar lavage fluid was collected of both lung halves with 20 ml PBS under general anesthesia. Day 3 post-challenge pigs were euthanized by electroshock and subsequent exsanguination. Sampling was performed immediately after exsanguination and included swabs, blood for serum, and organs for RT-PCR and histology.

### Virological Analyses

Of each animal, all four cranial lobes and a bronchial lymph node were collected into 1.5 ml tubes containing 500 μl MEM medium (Life Technologies) and weighed before lysing with a BulletBlender^®^ (Next Advanced Inc., Averill Park, NY, USA). Lysed organs were centrifuged, and the supernatants transferred into new tubes and stored −70°C. RNA was extracted using the QIAmp^®^ viral RNA extraction kit (Qiagen AG, Hombrechtikon, Switzerland) according to manufacturer’s instructions and amplified by quantitative reverse transcriptase polymerase chain reaction (RT-qPCR) using primers and probe, as described (17, 18). The RT-qPCR was performed as published using the SuperScript^®^ III Platinum^®^ One-Step RT-qPCR Kit (Life Technologies) and run on a 7900HT Thermocycler (Applied Biosystems) for 50 cycles. Mean Cq values were determined from triplicates. For absolute RNA quantification, an internal standard was used based on M1 genome copies (17, 18).

### Serological Assays

For virus neutralization tests (VNT), sera were serially diluted in triplicates starting at a 1:5 dilution in virus growth medium (MEM, supplemented with HEPES and TPCK trypsin). One hundred TCID_50_/well of IAV were added, and the mix was gently agitated and incubated at 37°C for 30 min. Confluent MDCK cells were then incubated with the serum-virus mix at 37°C, and after 24 h, 100 μl medium was added in each well. After another 24 h, the cells were fixed and stained as described earlier, and VNT titers were read as the last serum dilution that prevented infection. As anti-NA antibodies do not neutralize but prevent cell-to-cell spread, the titer of sera obtained from animals immunized with NA only was defined as the last dilution able to inhibit viral spread resulting only in single cell infections. Plaque reduction assays were performed as VNTs with the following modifications. Only 50 μl of serum/virus mix was added to MDCK cells and incubated for 6 h at 37°C. Then, 150 μl of 0.8% methylcellulose (Sigma-Aldrich) medium were added to each well and incubated for another 36 h before harvest.

To identify antibodies reactive with HA or NA expressed on the surface of infected cells, MDCK cells were infected for 12 h with IAV, fixed, and stained with the immune sera followed by an fluorochrome-conjugated goat anti-pig antibody (Bethyl, Montgommery, TX, USA) and analyzed by flow cytometry (FACSCalibur, BD Biosciences, Allschwil, Switzerland).

For opsonization assays, we made use of a previously established assay based on the ability of plasmacytoid dendritic cells (pDCs) to secrete enhanced INF-α in response to immune complexed virus compared with free virus *via* an Fcγ receptors (FcγR)-dependent pathway (19–21). To this end, peripheral blood mononuclear cells (PBMCs) were freshly isolated from blood of our SPF pigs using ficoll-paque density centrifugation (1.077 g/l, Amersham Pharmacia Biotech). pDCs were enriched by cell sorting of CD172a^+^ PBMCs using the magnetic cell sorting system (MACS) with LD columns (Miltenyi Biotec GmbH, Germany), as described previously (19). This permits a 10- to 20-fold enrichment of pDC. IAV were then first titrated on pDC to find virus doses inducing low levels of pDC activation by the virus alone. As this is highly variable with different strains of IAV (22), the optimal doses were determined with each virus isolate and for each virus stock to be employed. Then, sera were serially diluted in DMEM supplemented with 1% porcine SPF serum and 50 μM β-mercaptoethanol, followed by incubation with the determined viral dose and incubated for 20 min at 39°C. Enriched pDCs were then added, and the cultures incubated for 16 h at 37°C. Supernatants were tested for IFN-α by ELISA (19). Virus opsonizing titers were defined as the highest serum dilution resulting in a significantly higher IFN-α production compared with naive serum.

### Pathology

For the macroscopic scoring, pneumonic lesions characterized by increased redness and consolidation of lung tissue were scored according to the following scheme: ventral and dorsal aspects of the cranial lobe received a maximum of 5 points each, ventral aspects of the caudal lobes a maximum of 12.5 points, dorsal aspects of the caudal lobes 15 points, and accessory lobes (ventral aspects only) a maximum of 5 points. All points were summed up to a total macroscopic score. For histological examination, cranial lobes were fixed in 10% buffered formalin, processed routinely for paraffin embedding, cut at 5 μm, and stained with hematoxylin and eosin (H&E). A board certified pathologist scored all histological sections in a blinded manner. Bronchi and bronchioles were scored for epithelial necrosis, fibrin exsudation, and infiltration with neutrophils. Alveoli were scored for epithelial necrosis, fibrin exsudation, hemorrhage, neutrophilic infiltration, thickening of the alveolar walls, and atelectasis. The following scores were given to each of the abovementioned findings: 0 = no lesions; 1 = very mild lesions (<5% of structures affected), 2 = mild lesions (5–20% of structured affected), 3 = moderate lesions (20–40% of structures affected), 4 = severe lesions (40–60% of structured affected), and 5 = very severe lesions (more than 60% of structures affected). All scores were summed up to a total microscopic lung score.

### T Cell Assays

Peripheral blood mononuclear cells were thawed and cultured in AIM medium Albumax (Gibco) with 2% FBS and stimulated with IAV (Belzig/01 or R757/10) MOI 0.1 TCID_50_/cell or a corresponding volume of chicken allantoic fluid (CAF) for 15 h at 39°C. Brefeldin A (ebioscience, San Diego, CA, USA) was added for another 4 h before harvest. Cells were first stained with Fixable Aqua Dead Cell Stain kit (ThermoFisher, Waltham, MA, USA). Then, surface staining employed anti-CD4 IgG2b (74-12-4) and anti-CD8β IgG2a (PG164A; VMRD, Pullmann, WA, USA), followed by isotype-specific Alexafluor-488 and PE-Cy7 fluorochrome conjugates (Thermofisher and Abcam, respectively). After fixation and permeabilization, anti-IFNγ-PE (P2G10, BD Biosciences) and anti-TNFα-AF647 (Mab11, Biolegend, San Diego, CA, USA) were added. Cells were analyzed by flow cytometry (FACSCanto). Dead cells were excluded, followed by doublet discrimination, and gating on CD4 and CD8 and single positive cells to determine their intracellular IFNγ and TNFα expression.

### Statistical Analysis

For multiple group comparisons one-way ANOVA, for pair comparisons two-way Mann–Whitney *U* test, and for non-pair comparisons Wilcoxon test were used. *p* < 0.05 was considered significant. The Graph Pad Prism software (GraphPad software, La Jolla, CA USA) was employed.

## Results

### Neutralizing Activity and Cross-Reactivity of Antibodies Induced by VRPs Expressing H1 and N1

The first aim of this study was to characterize anti-HA and anti-NA antibodies with respect to their neutralizing activity against various IAV. To this end, we immunized pigs with VSV*ΔG(H1) or VSV*ΔG(N1) VRPs expressing the HA and NA antigens of A/swine/Belzig/2/01 (H1N1) (Belzig/01). Control animals received the VSV*ΔG VRP only expressing GFP but not any influenza antigen. As a reference vaccine, WIV prepared from a Belzig/01 was used. None of the animals showed any adverse effects in response to the VRP vaccines, but the two pigs vaccinated with WIV had a transient increase of body temperature by 0.5°C for 1 day, both following prime and booster injection.

**Figure 1:**
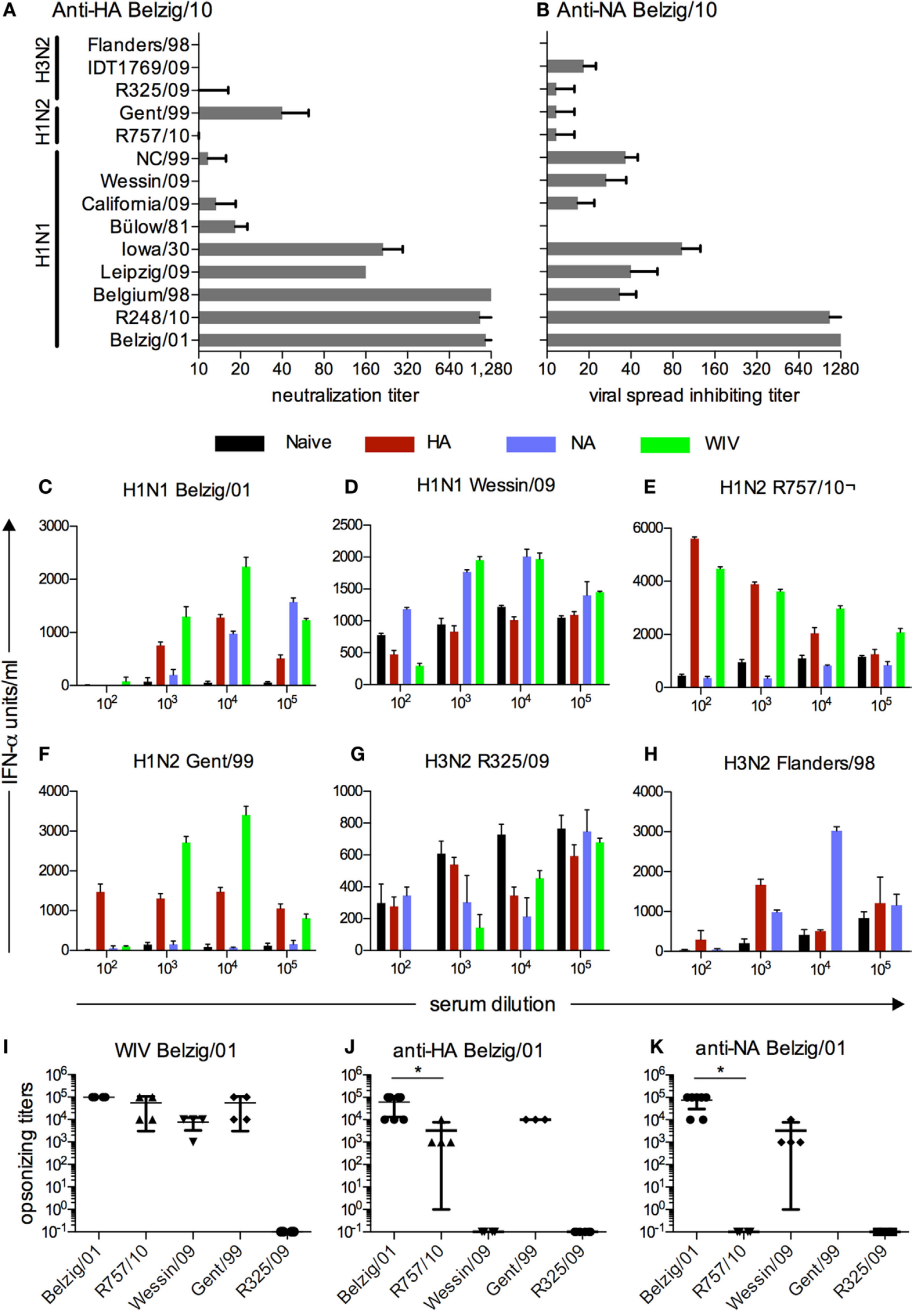
**Functional characterization and cross-reactivity of sera from vaccinated pigs**. **(A)** Neutralizing activity against various IAV of anti-HA sera from pigs that were vaccinated with VSV*ΔG(H1) of Belzig/01. **(B)** Inhibitory activity of anti-NA sera from pigs vaccinated with VSV*ΔG(N1) of Belzig/01 against virus spread in cell culture. **(B)**. In **(A,B)**, titers were defined as the last serum dilution that inhibited infection or allowed only single cell infection. The sera were collected 3 weeks after the second booster immunization. Mean and SD of six replicates from a representative experiment out of two is shown. **(C–H)** Different viruses were tested in a pDC-based opsonization assay based for antibody-enhanced pDC activation and IFNα secretion following stimulation with immune complexes. Sera from naive, VSV*ΔG(H1), VSV*ΔG(N1), and WIV vaccinated pigs were serially diluted and tested for their ability to enhance IFNα of pDC stimulated by various IAV. A representative experiment is shown. **(I–K)** Mean and SD of all pDC-based opsonization assays performed. Opsonizing titers were defined as the highest serum dilution resulting in a significantly higher IFN-α production by enriched pDC as compared with naive serum. Each symbol represents an independent experiment. Asterisks (*) indicate significant differences calculated with the Wilcoxon test (*p* < 0.05).

The immune sera from vaccinated pigs obtained 3 weeks after the booster vaccination were analyzed for both neutralizing and opsonizing activity in order to identify a virus, which could be opsonized but not neutralized. To this end, we first performed VNTs with a collection of porcine and human H1N1 IAV as well as porcine H1N2 and H3N2 IAV (Figure 1A). Sera from pigs vaccinated with VSV*ΔG(H1) had titers of 1:1280 against the homologous virus and two others H1N1 viruses of the same subgroup (R248/10 and Belgium/98). The titers for Leipzig/09 and Iowa/30 were 160, while all other viruses were not or only weakly neutralized including two isolates from the 2009 pandemic (Wessin/09 and California/09). A low level of neutralization was also found against Gent/99 (H1N2). These results were confirmed by a plaque reduction test (data not shown).

Sera obtained from pigs vaccinated with the VSV*ΔG(N1) were tested for their ability to inhibit viral spread using the same collection of viruses (Figure 1B). Spread of the homologous virus and R248/10 was still inhibited at a serum dilution of 1:1280. Overall, anti-N1 sera showed a broader reactivity against different N1 viruses with the exception of Bülow/81, which was not inhibited. N2 viruses were not inhibited by anti-N1 serum with the notable exception of IDT1769/09, which was inhibited to some extent.

### Opsonizing Activity of Anti-H1 and Anti-N1 Antibodies

Our next aim was to identify viruses, which were opsonized by immune sera in the absence of virus neutralizing activity. To this end, an assay was employed that was based on the ability of immune serum to enhance IAV-induced INF-α secretion from pDCs. The anti-H1, anti-N1, and anti-WIV sera were all able to opsonize the homotypic virus at dilutions ranging from 10^−3^ to 10^−5^ (Figure 1C). The heterotypic Wessin/09 (H1N1) was not opsonized by anti-H1 sera, but was opsonized by sera from pigs vaccinated with VSV*ΔG(N1) and WIV at dilutions ranging from 10^−3^ to10^−4^ (Figure 1D). Using the H1N2 viruses R757/10 and Gent/99, enhanced INF-α was found with serum from pigs vaccinated with VSV*ΔG(H1) or WIV, but not with anti-N1 serum. The serum dilutions were ranging from 10^−2^ to 10^−5^ (Figures 1E,F). The two H3N2 viruses tested showed different phenotypes. While R325/09 was not opsonized by any serum (Figure 1G), Flanders/98 was opsonized by anti-H1 and anti-N1 sera at dilutions of 10^−3^ and 10^−4^, respectively (Figure 1H). With some of the viruses, “bell-shaped” titration curves of the opzonizing titers were found. A possible explanation for the lower INF-α at the lower 10^−2^ dilutions could be inhibitory effects of antibodies preventing endosomal fusion of the virus, resulting in reduced sensing by the cells. These antibody specificities could be diluted out along the titration.

These opsonization experiments were repeated several times in independent experiments, and the results are shown in Figures 1I–K for the sera obtained from the different vaccines. Based on these results, we selected the R757/10 for challenge infection experiments as this virus was efficiently opsonized but not neutralized by sera generated against H1 and WIV, while anti-NA serum did not show and cross-reactivity. We confirmed the ability of anti-H1 to bind to R757/10 using infected MDCK cells. Antisera directed to Belzig/01 HA bound to both Belzig/01- and R757/10-infected cells, whereas sera directed to Belzig/01 NA reacted exclusively with Belzig/01-infected cells (Figure S1 in Supplementary Material).

### Kinetics of Antibody Responses Induced by VSV-Based VRP Vaccines

**Figure 2:**
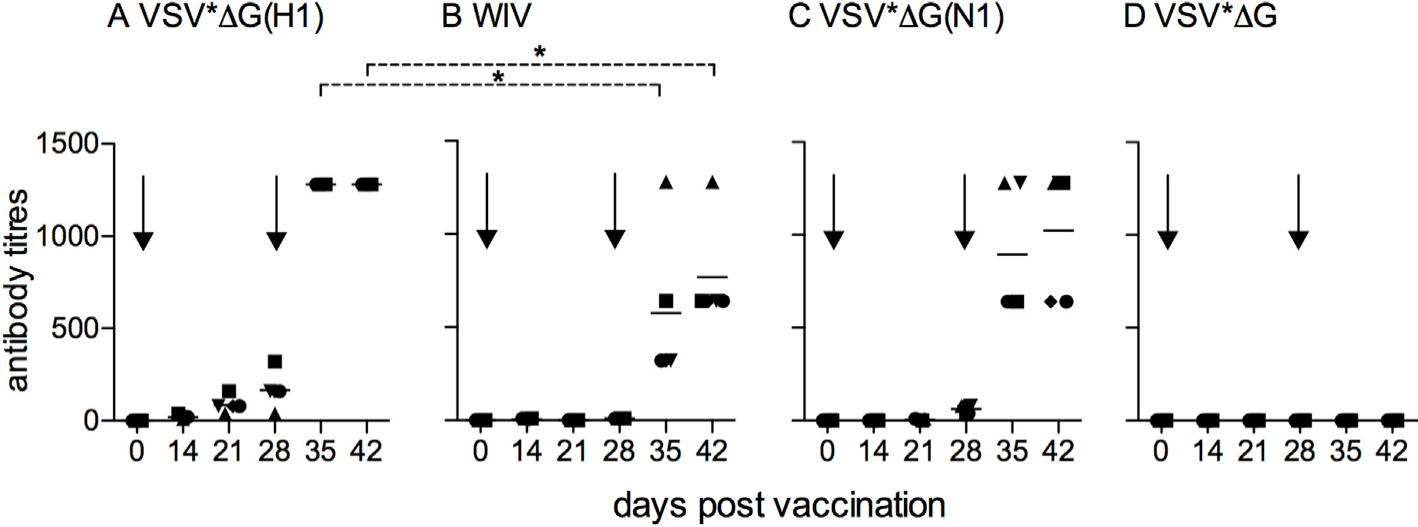
**Kinetics of antibody responses induced by VSV-based VRP vaccines**. Sera from pigs (five animals per vaccine group) that were vaccinated with **(A)** VSV*ΔG(H1), **(B)** VSV*ΔG(N1), **(C)** VSV*ΔG, or **(D)** WIV were collected at the indicated time points, and tested for neutralization **(A,C,D)** or inhibition of viral spread **(B)** against homologous virus (Belzig/01). At day 28, all animals received a booster immunization. Arrows indicate the vaccination time points. Each symbol represents a different animal. Asterisks indicate significant differences between groups at particular days post-vaccination calculated with the Wilcoxon test (*p* < 0.05).

We next vaccinated pigs in groups of five with either VSV*ΔG(H1), VSV*ΔG(N1), or WIV, with all antigens derived from Belzig/10. A control group of five animals was vaccinated with VSV*ΔG, and three animals were not injected. Following a booster injection at day 28, VSV*ΔG(H1) and WIV vaccinated animals developed good Belzig/01-neutralizing antibody titers and VSV*ΔG(N1) antibodies that inhibited Belzig/01 spread. VSV*ΔG(H1) induced the highest levels of neutralizing antibodies in all five animals of the group, whereas only one pig from the WIV-vaccinated vaccinated group reached similar high levels of neutralizing antibodies. No anti-IAV immune response was found in pigs vaccinated with VSV*ΔG (Figures 2A–D).

### Body Temperature and Pathology Following Heterologous Challenge

**Figure 3:**
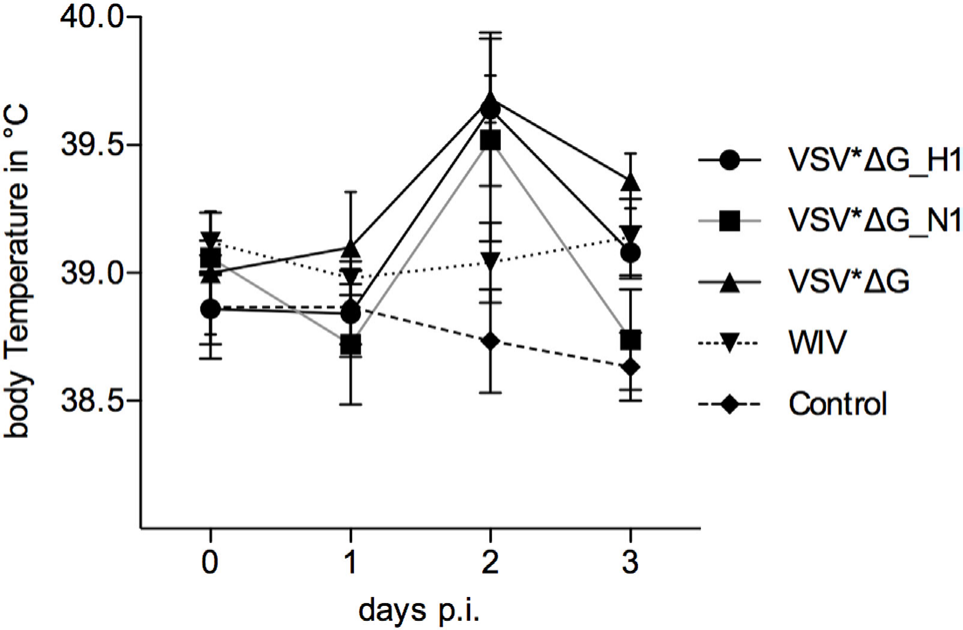
**Body temperature following heterologous challenge with R757/10**. VSV*ΔG(H1)-, VSV*ΔG(N1)-, VSV*ΔG-, or WIV-vaccinated animals were challenged with R757/10 (H1N2). In **(A)**, mean values and SDs of body temperatures are shown for each group. In **(B)**, values for individual animals are shown for day 2 p.i.

At day 42 post-vaccination, pigs were infected with R757/10. No coughing or increased breathing was observed, and only a mild, transient increase in body temperature was recorded (Figure 3). On day 2 p.i., the mean body temperature in the VSV*ΔG(H1), VSV*ΔG(N1), and VSV*ΔG groups was higher than the mean body temperature of non-infected pigs. Nevertheless, statistical analysis did not reveal significant differences between the groups.

**Figure 4:**
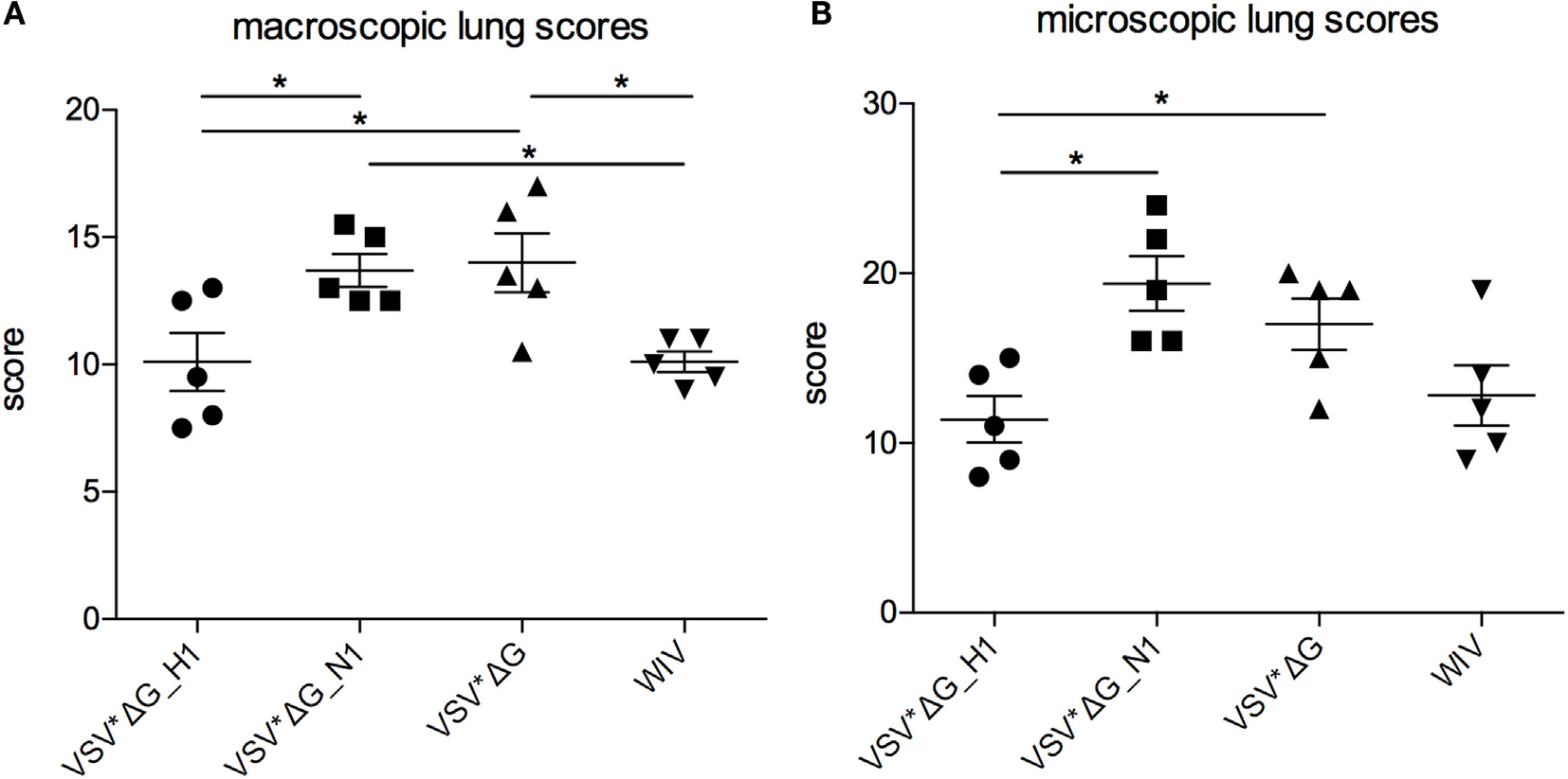
**Lung lesions after heterologous challenge with R757/10**. Lung lesions were assessed **(A)** macroscopically and **(B)** microscopically from each lobe at 3 days p.i. Scores were determined by a pathologist who was blinded for the treatments and assessed for each lobe. The values were pooled to one value per animal. Asterisks indicate significant differences calculated with the Wilcoxon test (*p* < 0.05).

Three days p.i., all animals were slaughtered and the lungs were analyzed macroscopically and histologically. Generally, pathological lesions were mild reaching a maximum macroscopic lung score value of 18/100. Nevertheless, the macroscopic lung alterations were significantly more severe in animals vaccinated with VSV*ΔG(N1) or VSV*ΔG than in pigs vaccinated with VSV*ΔG(H1) or WIV (Figure 4A). Histological analysis confirmed mild lesions in the lungs of animals with the highest macroscopical lung scores (Figure 4B). The histopathological findings were typical for influenza virus-induced lesions of an acute broncho-interstitial pneumonia. Lesions were mainly located in the bronchiole and characterized by epithelial necrosis, mild fibrin exsudation and filling of bronchiole with neutrophils. Adjacent alveoli were atelectatic or filled with erythrocytes, fibrin, and neutrophils (Figure S2 in Supplementary Material). Animals vaccinated with VSV*ΔG(H1) had significantly lower histological lung scores when compared with animals that had received either VSV*ΔG or VSV*ΔG(N1).

### Viral RNA Loads Following Challenge Infection

**Figure 5:**
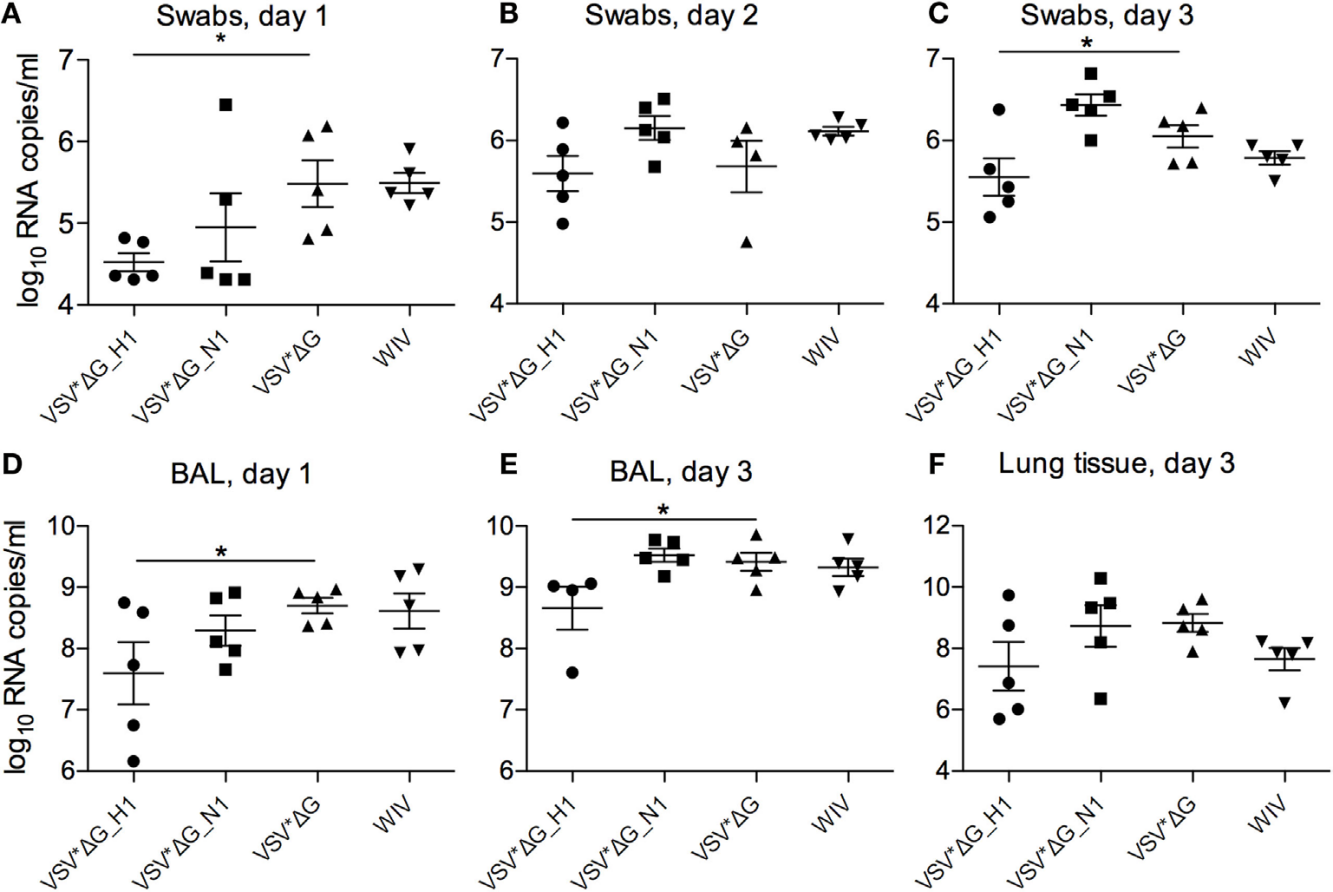
**Viral RNA loads after heterologous challenge with R757/10**. Viral RNA copies per milliliter were assessed by RT-qPCR in swabs on days 1 **(A)**, 2 **(B)**, and 3 **(C)**, in the BAL fluid on day 1 **(D)** and day 3 **(E)**, and in the lung tissue on day 3 **(F)**. Asterisks (*) indicate significant differences calculated with the Wilcoxon test (*p* < 0.05).

At day 1 p.i., viral RNA quantities in oronasal swabs were significantly lower in animals vaccinated with VSV* ΔG(H1) compared with those vaccinated with VSV*ΔG (*p* = 0.02) (Figure 5A). The other three groups did not significantly differ from the VSV* ΔG control group at day 1 p.i. On day 2, no differences between the groups could be observed; however, at day 3 p.i., the VSV*ΔG(H1)-vaccinated animals showed significantly lower viral loads (Figures 5B,C; *p* = 0.047). Likewise, in bronchoalveolar lavages, viral RNA copies were lower at day 1 p.i. if the animals had been vaccinated with VSV*ΔG(H1) as compared with the VSV*ΔG control group (Figure 5D; *p* = 0.042). On day 3 p.i., viral load increased in all groups but remained lower in H1-vaccinated animals (Figure 5E; *p* = 0.03). In lung tissue (Figure 5F) as well as in the draining bronchial lymph node (data not shown), no significant differences were observed between the four vaccine groups.

### Cytokine Responses Following Challenge Infection

**Figure 6:**
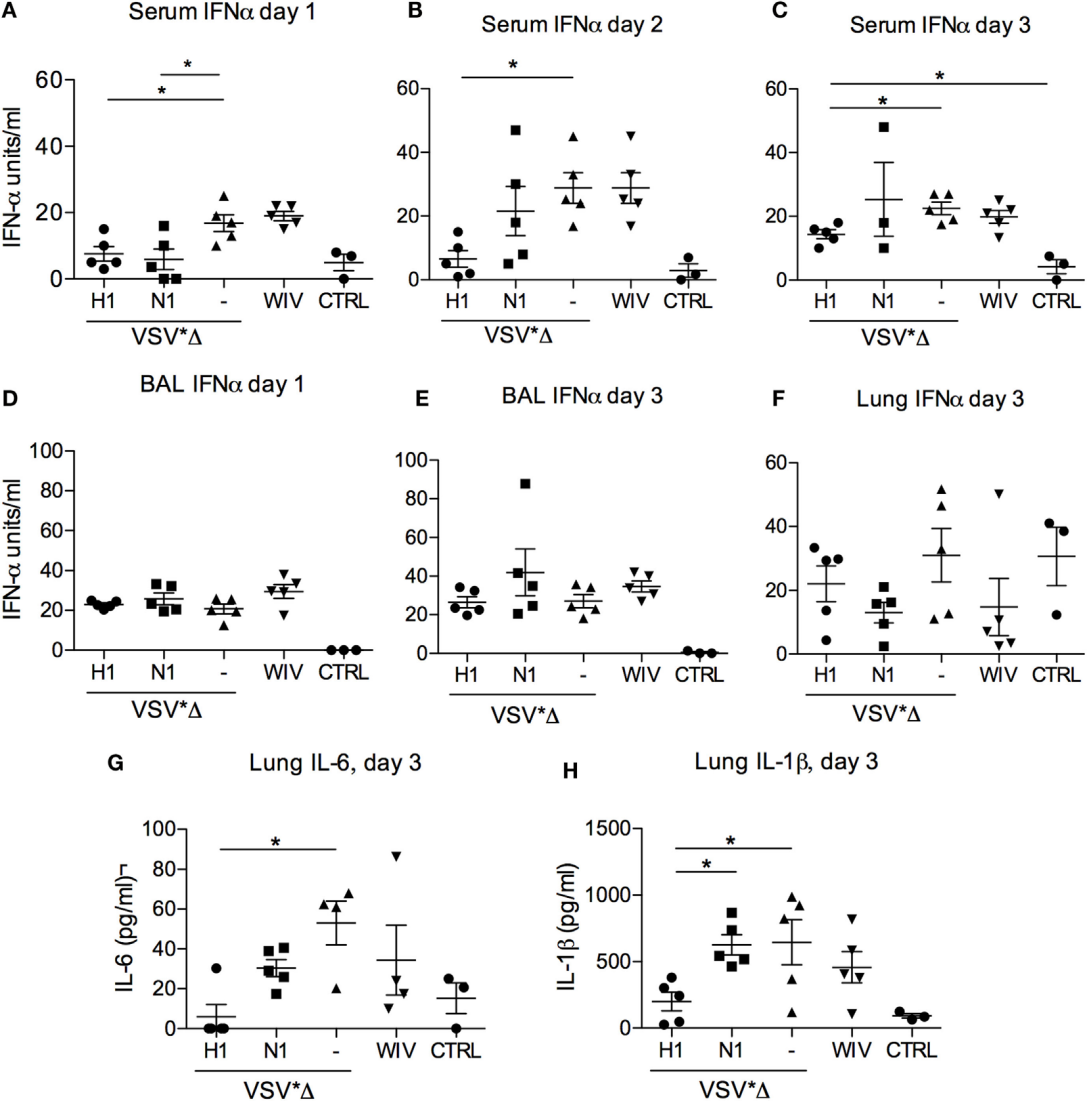
**Innate cytokine responses after heterologous challenge with R757/10**. In **(A–C)** IFNα levels were determined by ELISA in the serum of the infected animals on days 1 **(A)**, 2 **(B)**, and 3 **(A)** post-infection. **(D,E)** show the IFNα levels in the BAL on day 1 and 3, respectively. In **(F–H)** cytokine levels were determined in lung tissue lysates obtained on day 3 post-challenge. “CTRL” stands for samples obtained from non-infected SPF pigs from the same breeding. Each symbol represents a different animal. Asterisks (*) indicate significant differences calculated with the Wilcoxon test (*p* < 0.05).

As local and systemic inflammatory cytokine and IFN-α responses may also reflect levels of virus replication, we analyzed such responses as an additional indicator of vaccine-induced protection (23, 24). Altogether, relating to the relatively mild disease, systemic IFN-α remained at low levels during the whole observation period (Figure 6). At day 1 p.i., all animals showed only low levels of IFN-α; however, in both H1- and N1-vaccinated animals, the levels were significantly lower than in the VSV*ΔG control group (Figure 6A; *p* = 0.022). At days 2 and 3 p.i., only the VSV*ΔG(H1)-vaccinated animals stayed at significantly lower cytokine levels than the control group (Figure 6B; *p* = 0.002 and *p* = 0.01, respectively). Nevertheless, IFN-α was not completely controlled as serum levels in VSV*ΔG(H1)-vaccinated pigs that were significantly higher than those in non-infected controls at day 3 p.i. In BAL collected at days 1 and 3 p.i. and also in lung tissue, no significant differences in IFN-α levels were observed between all groups (Figures 6D–F). In contrast to this, significant differences were found in lung tissue with respect to inflammatory cytokines. VSV*ΔG(H1)-vaccinated pigs had significantly lower levels of IL-6 when compared with the VSV*ΔG control group (*p* = 0.01) (Figure 6G). The IL-1β levels in the VSV*ΔG(H1) group were significantly lower than those in the VSV*ΔG (*p* = 0.02) and VSV*ΔG(N1) group (*p* = 0.04) (Figure 6H). Neither IL-1β nor IL-6 was increased in the serum and BAL fluid (data not shown).

### VRP but Not WIV-Induced Dual-Functional CD4 Th Cells Secreting IFNγ and TNFα

**Figure 7:**
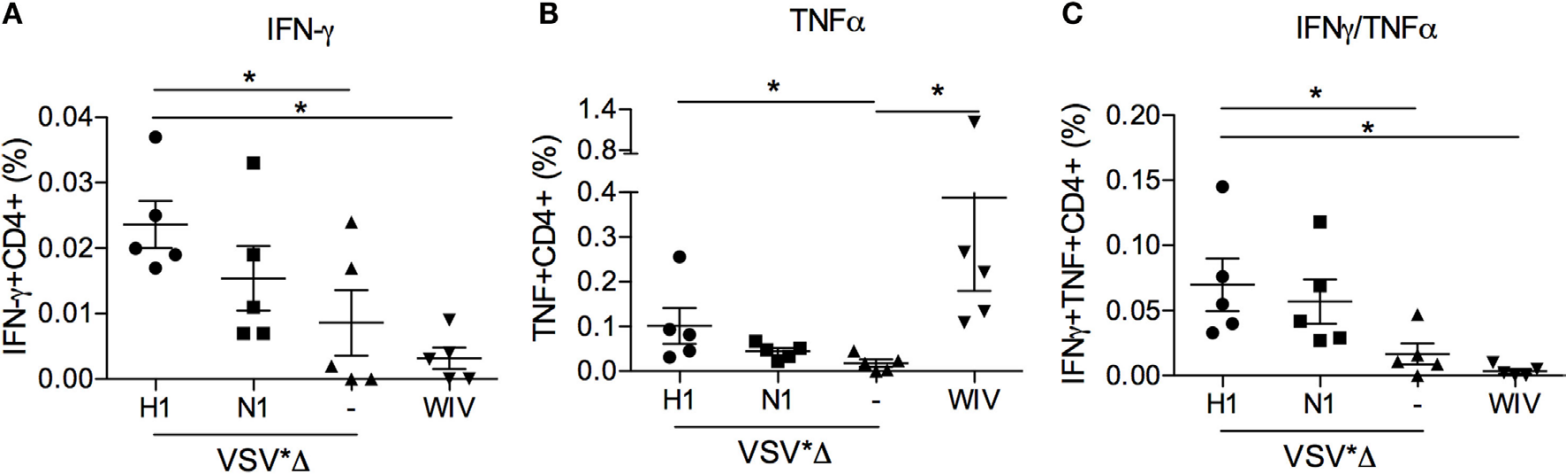
**Peripheral blood CD4 T-cell responses induced by VSV-based VRP and WIV vaccines**. PBMCs were isolated at 3 weeks post-booster vaccination and restimulated with chicken allantoic fluid (CAF) as a mock control or IAV *in vitro* to determine the percentage of virus-specific IFNγ- **(A)**, TNFα- **(B)**, and dual cytokine **(C)** producing cells in the CD4 T cell subset. The percentage of virus-specific cytokine producing cells was determined by subtracting the values of the CAF controls from the IAV-stimulated cultures. The gating strategies and CAF/IAV percentages are shown in the Figure S3 in Supplementary Material. Asterisks (*) indicate significant differences calculated with the two-way Mann–Whitney *U* test (*p* < 0.05).

Given that both the VSV*ΔG(H1) and the WIV vaccine-induced opsonizing antibodies reactive with the challenge virus while only the VSV*ΔG(H1) vaccine-induced partial protection, we questioned if this would be related to differences in the ability to induce T-cell responses. Therefore, PBMCs collected 3 weeks post-booster vaccination were restimulated *in vitro* with IAV, and CD4 and CD8 T cell subsets were analyzed for their ability to produce IFNγ and TNFα using multicolor flow cytometry (see Figure S3 in Supplementary Material for gating strategy and raw data). In Figure 7, virus-specific CD4 T cell responses are shown. In animals vaccinated with WIV, only IAV-specific TNFα-producing CD4^+^ T cells were found (*p* = 0.008). In contrast, stimulation of PBMC that were collected from the VSV*ΔG(H1) vaccine groups resulted in a significant production of IAV-specific IFNγ (*p* = 0.03), TNFα (*p* = 0.02), and dual IFNγ- and TNFα-producing (*p* = 0.03) CD4^+^ T cells. PBMC from VSV*ΔG-vaccinated control animals did not show any significant IAV-specific reaction upon restimulation (Figure S3 in Supplementary Material). No response was detected in the CD8^+^ T cell subset in any of the groups. We also tested PBMC prior to vaccination and found no IAV-specific IFNγ or TNF response (data not shown).

Taken together, our results indicate that only VSV*ΔG(H1) vaccinated animals were partially protected (summarized in Table 2). This was based on the ability of the vaccine to reduce macroscopic and microscopic lung lesions, viral RNA loads in swabs and BAL fluids, systemic IFNα responses, and local inflammatory cytokine induction, although the viral loads in the lungs were not significantly reduced. A possible explanation for this discrepancy could be that, by chance, we picked areas the lung with little virus replication, as virus load can greatly vary in different regions of the pig lung. The immune responses correlating with partial protection were characterized by the induction of opsonizing antibodies and the presence of CD4 T helper cells in the peripheral blood, which secreted both IFNγ and TNFα.

**Table 2:** Summary of vaccine-induced effects compared with VSV*ΔG.

	VSV*ΔG_HA	VSV*ΔG_NA	WIV
Body temperature	None	None	*P* = 0.019
Macroscopical lung lesions	Reduction	None	Reduction
Histological lung lesions	Reduction	None	None
Viral RNA loads in swabs	Reduction	None	None
Viral RNA loads in BAL	Reduction	None	None
Viral RNA loads in lung tissue	None	None	None
IFNα in serum	Reduction	None	None
IFNα in BAL	None	None	None
IL-6 in lung tissue	Reduction	None	None
IL-1β in lung tissue	Reduction^a^	None	None

^a^When compared with VSV*ΔG_NA; p = 0.04.

## Discussion

Due to constant antigenic drift and occasional antigenic shift, the continuous selection of vaccines matching circulating strains represents a critical issue with respect to vaccine-induced protective immunity. This is further complicated by recent observations in human and pigs demonstrating that pre-existing immunity lacking neutralizing antibodies can enhance respiratory disease. In human, enhanced disease was first described with the emergence of the pandemic H1N1 strain in 2009. Affected patients were characterized by the presence of high titers of antibodies that bound to the pandemic strain but were unable to neutralize it, and this was associated with pulmonary deposition of immune complexes (11). Furthermore, reports from Canada indicated that patients who were vaccinated in 2008/09 with WIV had an increased risk of severe illness if they were infected with pandemic H1N1 in 2009 (25). Similar observations were made with Chinese patients in 2009. Severe influenza disease in these patients was apparently related to high levels of non-neutralizing antibodies as detected by ELISA (9). After these reports had been published in 2010, we initiated our studies with the aim to investigate the role of non-neutralizing opsonizing antibodies against HA in the pig model. Opsonizing antibodies were detected according to their ability to enhance cytokine responses in pDC. The evaluation of clinical, virological, and immunological parameters demonstrated that the WIV vaccine had no protective effect, neither did it enhance disease. In contrast, the VSV*ΔG(H1) VRP vaccine-mediated partial protection. As both vaccine types induced opsonizing antibodies, we conclude from these findings that opsonizing antibody responses can neither be taken as a correlate of enhanced disease nor of partial protection.

Our study appears to be in contrast with a series of publications describing enhanced disease in pigs that were vaccinated with a H1N2 WIV and challenged with a 2009 pandemic H1N1 strain. The authors found that vaccination with WIV potentiated clinical signs, lung inflammation, and release of inflammatory cytokines but had no effect on viral loads (7, 8). It is also important to note that enhanced disease was reported with challenge viruses other than virus strains from the 2009 pandemic (26, 27). Furthermore, other groups have employed a 2009 pandemic strain as heterologous challenge virus and found no evidence for enhanced disease (28). Interestingly, enhanced disease has been observed only with WIV and subunit HA vaccines but was not observed with live attenuated virus vaccines and an HA-recombinant adenovirus 5 vector vaccine (8, 27, 29), suggesting that enhanced immunity may occur if a mismatched vaccine lacks sufficient priming of T cell-mediated immunity. Furthermore, a recent study demonstrated that enhanced diseases were only found when the NA was mismatched and thus not able to contribute to protection (30). Nevertheless, enhanced disease seems to be a relatively rare event occurring only with a particular strain of porcine IAV. The present study and other vaccine studies performed with WIV in pigs found no enhanced disease despite the absence of vaccine-induced neutralizing or hemagglutination-inhibition antibodies (31, 32).

Non-neutralizing opsonizing antibodies have not only been associated with enhanced disease, they have also been demonstrated to possess protective potential. In ferrets and mice, cross-protection was observed through cross-reactive but non-neutralizing antibodies (33). On the one hand, heterologous immunity was abrogated in B cell-deficient mice but maintained in CD8^−/−^ and perforin^−/−^ mice. On the other hand, passive transfer of immune serum conferred protection in naive recipient mice when subsequently challenged with the 2009 pandemic H1N1 virus (33). Furthermore, broadly neutralizing monoclonal antibodies against the conserved stalk region of HA required FcR to confer protection in a mouse model, an observation not found with antibodies directed against the variable head domain of HA (34).

An important finding of our study is that the VSV*ΔG(H1) VRP vaccine provided better protection than WIV. On the one hand, the VRP vaccine induced higher levels of antibodies compared with WIV, at least in terms of homologous neutralization. On the other hand, only after vaccination with the VRP vaccine antigen-specific CD4 T helper cells secreting both IFNγ and TNFα could be detected in the blood. CD4 T cell responses play an important role in the protection against IAV (35, 36). For example, in mouse models, they are able to confer protection independently of B cells and CD8 T cells (37), and in humans, protection was correlated with preexisting influenza-specific CD4 T cells (38). Interestingly, CD4 T cells directed against HA, but not NP, were found to correlate well with the development of neutralizing antibody responses (36, 39). Although HA is much less conserved than NP, CD4 T cells primed during an H1 virus infection were found to react with even different subtypes, such as H5 and H7 (40, 41). Finally, multifunctionality of CD4 T cells has also been correlated with protective immune responses (42). All these previous findings are in line with the possibility that the partial protection against mismatched virus was induced by the superior activation of IAV-specific CD4 T cells by VRP vaccine.

In summary, this work demonstrates that WIV vaccines, which induce opsonizing antibodies lacking neutralizing activity, induce enhanced disease only in certain conditions, which are not yet well understood. Furthermore, we demonstrate with the virus pair selected that VSV-based VRP vaccines previously used successfully for protection of chickens from infection with avian IAV (14, 43) are also promising in swine as they induced both antibodies and T-cell immunity.

## Author Contributions

MR and AS conceived the idea, designed the study, acquired and analyzed data, and wrote the manuscript. NV, SP, BZ, DB, and HP acquired and analyzed data, and reviewed the manuscript. GZ designed the study, helped with interpretation of the data, and reviewed the manuscript. All authors gave their final approval for submission.

## Conflict of Interest Statement

The authors declare that the research was conducted in the absence of any commercial or financial relationships that could be construed as a potential conflict of interest.

## Supplementary Material

Figure S1 **Cross-reactivity of anti-Belzig/01 HA sera with cell surface expressed R757/10 HA**. Flow cytometry histogram plots demonstrating the binding of sera from VSV*∆G(H1) and VSV*∆G(N1) vaccinated pigs to Belzig/01 and R757/10 infected MDCK. Similar results were obtained with sera from a second vaccinated pig.

Figure S2 **Histological lesions induced by R757/10 challenge**. Histological sections (overview left, higher magnification right) of representative lung samples showing no lesions **(A,B)**, very mild **(C,D)**, mild **(E,F)**, or moderate **(G,H)** lesions typical for viral induced damage to bronchiole and resulting inflammatory reaction. Sections in **(A–D)** derived from a pig of the VSV*∆G(H1) group, whereas lesions depicted in **(E–H)** derived from VSV*∆G(N1) vaccinated pig. Asterisks mark bronchiole, arrows point out epithelial damage, neutrophilic inflammation, fibrin exsudation in bronchioli. Note that depending on the severity of the lesion, adjacent alveoli within the corresponding lobulus were either mainly unaffected **(C,D)** or showed accumulation of edema fluid and neutrophils **(E,F)** or were completely atelectatic **(G,H)**. Staining: H&E, Bars: 500 µm (left), 100 µm (right).

Figure S3 **Peripheral blood CD4 T-cell responses induced by VSV-based VRP and WIV vaccines**. PBMC were isolated at 3 weeks post-booster vaccination and restimulated with CAF (C) or IAV (V) *in vitro* to determine the percentage of virus-specific IFNγ-, TNFα-, and dual cytokine-producing cells in the CD4 and the CD8 T cell subsets. In **(A)**, the gating strategy for live cells and single cells and CD4 or CD8 cells is shown. On the left side of **(B–E)**, representative IFNγ/TNFα dotplots for each vaccination group are shown for CD4 T cells. On the right side of **(B–E)**, the percentages of IFNγ-, TNFα-, and dual cytokine-producing cells for all animals are shown. Each symbol and color represents an individual animal. Asterisks (*) indicate significant differences between CAV and virus, calculated with the two-way Mann–Whitney *u* test (*p* < 0.05). No differences were found with the CD8 T cells (data not shown).
