# Supplementary figures and images for: Partial Protection against Porcine Influenza A Virus by a Hemagglutinin-Expressing Virus Replicon Particle Vaccine in the Absence of Neutralizing Antibodies
Source: Front Immunol. 2016 Jun 30;7:253. doi: 10.3389/fimmu.2016.00253 (PMC4928594; doi:10.3389/fimmu.2016.00253)

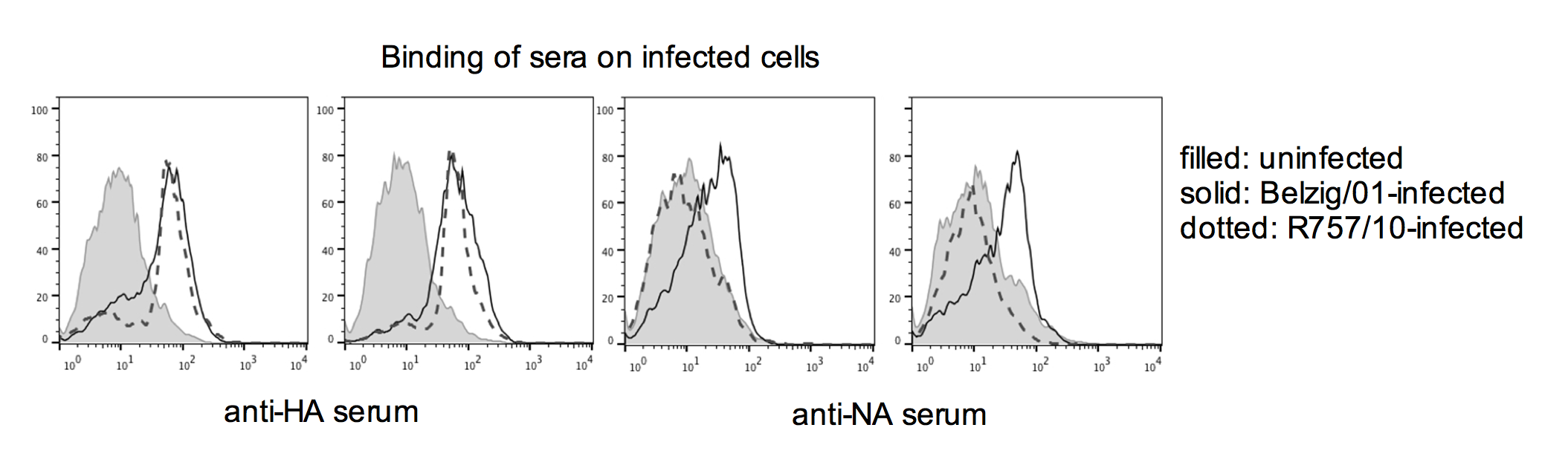

Supplement: Figure S1 — Cross-reactivity of anti-Belzig/01 HA sera with cell surface expressed R757/10 HA. Flow cytometry histogram plots demonstrating the binding of sera from VSV*∆G(H1) and VSV*∆G(N1) vaccinated pigs to Belzig/01 and R757/10 infected MDCK. Similar results were obtained with sera from a second vaccinated pig. [file Image_1.TIFF]

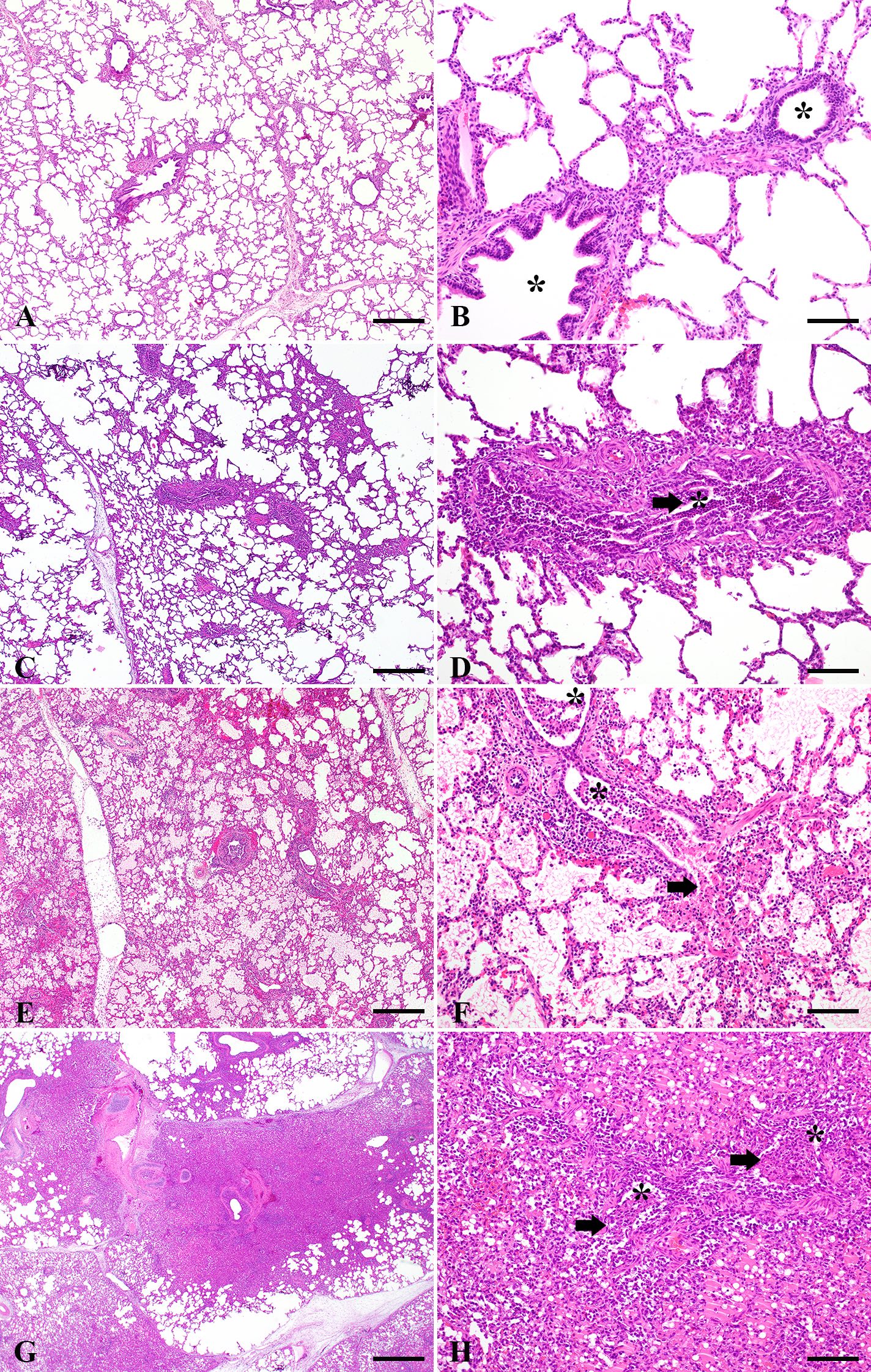

Supplement: Figure S2 — Histological lesions induced by R757/10 challenge. Histological sections (overview left, higher magnification right) of representative lung samples showing no lesions (A,B), very mild (C,D), mild (E,F), or moderate (G,H) lesions typical for viral induced damage to bronchiole and resulting inflammatory reaction. Sections in (A–D) derived from a pig of the VSV*∆G(H1) group, whereas lesions depicted in (E–H) derived from VSV*∆G(N1) vaccinated pig. Asterisks mark bronchiole, arrows point out epithelial damage, neutrophilic inflammation, fibrin exsudation in bronchioli. Note that depending on the severity of the lesion, adjacent alveoli within the corresponding lobulus were either mainly unaffected (C,D) or showed accumulation of edema fluid and neutrophils (E,F) or were completely atelectatic (G,H). Staining: H&E, Bars: 500 µm (left), 100 µm (right). [file Image_2.JPEG]

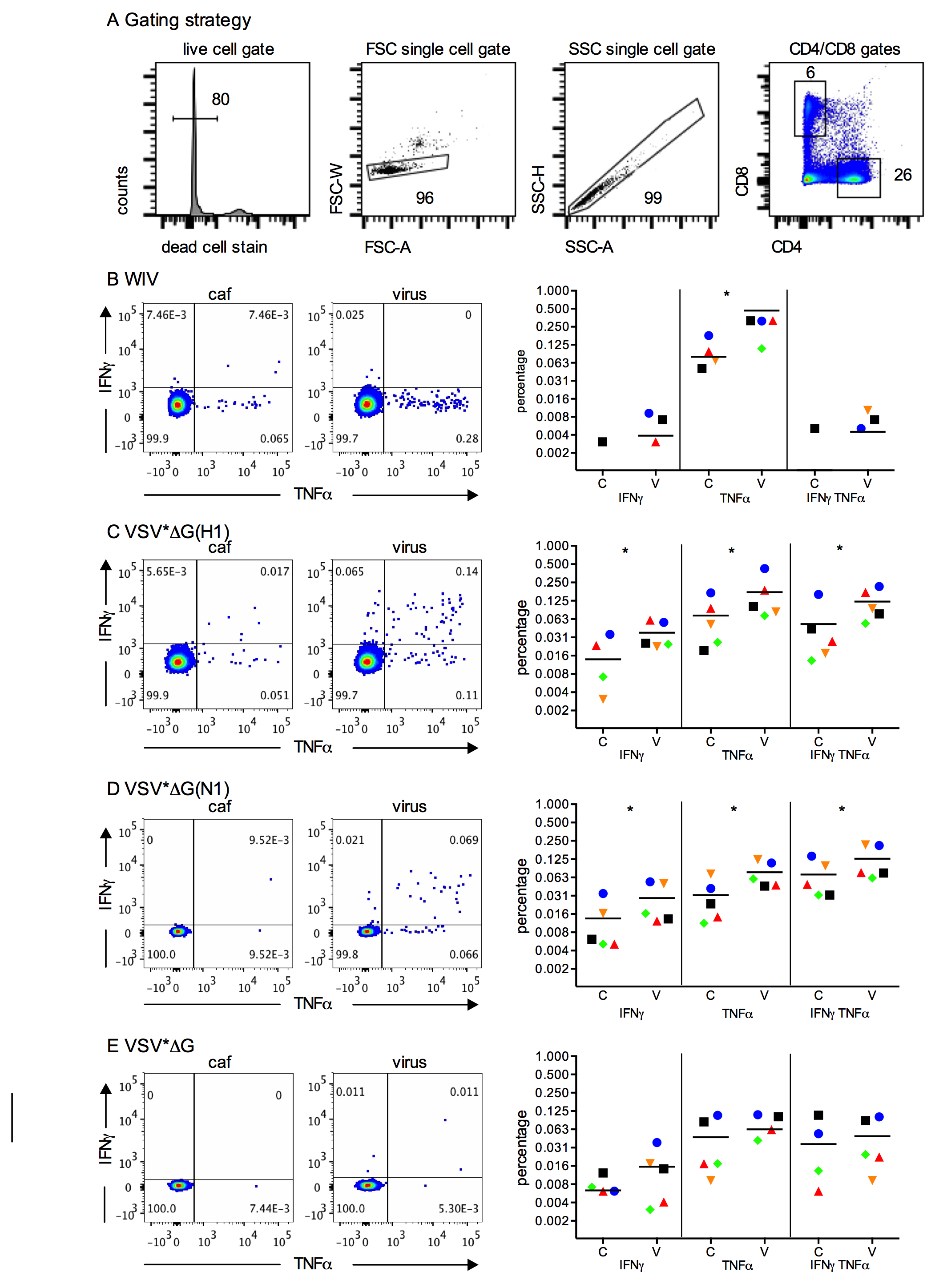

Supplement: Figure S3 — Peripheral blood CD4 T-cell responses induced by VSV-based VRP and WIV vaccines. PBMC were isolated at 3 weeks post-booster vaccination and restimulated with CAF (C) or IAV (V) in vitro to determine the percentage of virus-specific IFNγ-, TNFα-, and dual cytokine-producing cells in the CD4 and the CD8 T cell subsets. In (A), the gating strategy for live cells and single cells and CD4 or CD8 cells is shown. On the left side of (B–E), representative IFNγ/TNFα dotplots for each vaccination group are shown for CD4 T cells. On the right side of (B–E), the percentages of IFNγ-, TNFα-, and dual cytokine-producing cells for all animals are shown. Each symbol and color represents an individual animal. Asterisks (*) indicate significant differences between CAV and virus, calculated with the two-way Mann–Whitney u test (p < 0.05). No differences were found with the CD8 T cells (data not shown). [file Image_3.TIFF]
